# Cancer du sein triple négatif: particularités anatomo-cliniques et moléculaires

**DOI:** 10.11604/pamj.2022.42.30.28464

**Published:** 2022-05-12

**Authors:** Seifeddine Ben Hammouda, Nihed Abdessayed, Nouha Ben Abdeljelil, Hajer Hanchi, Dorra H´mida, Moncef Mokni

**Affiliations:** 1 Laboratoire d´Anatomie et Cytologie Pathologiques, Centre Hospitalier Universitaire Fattouma Bourguiba Monastir, Monastir, 5000, Tunisie,; 2 Laboratoire d´Anatomie et Cytologie Pathologiques, Centre Hospitalier Universitaire Farhat Hached, Sousse, 4031, Tunisie,; 3 Laboratoire de Cytogénétique, de Biologie Moléculaire et de Biologie de la Reproduction Humaines, Centre Hospitalier Universitaire Farhat Hached de Sousse, Sousse, 4031, Tunisie

**Keywords:** Cancer du sein, triple-négatif, biologie moléculaire, immunohistochimie, Breast cancer, triple-negative, molecular biology, immunohistochemistry

## Abstract

Le cancer du sein triple-négatif (CSTN) représente un sous-groupe de cancers du sein très hétérogène. L´objectif de notre étude était de détailler les caractéristiques anatomo-cliniques et moléculaires du CSTN et les comparer aux données de la littérature. Notre travail consistait en une étude rétrospective menée au service d´Anatomie et Cytologie pathologiques du CHU Farhat Hached de Sousse entre janvier 2018 et décembre 2020 ayant porté sur 56 cas de CSTN. Sur le plan épidémio-clinique, on a noté une incidence de 5,62% avec un âge moyen des patientes de 50,36 ans. La découverte d´un nodule du sein était le motif de consultation le plus fréquent (98,21%). Le diagnostic de CSTN était documenté dans 24 cas (42,86%) sur des pièces de mastectomie. Le type histologique le plus fréquent était le carcinome infiltrant de type non spécifique dans 47 cas soit 82,5 %. La taille tumorale moyenne était de 35,5 mm avec des extrêmes de 2 et 120 mm. Le grade SBR III était le plus prédominant dans notre étude (78,6%). Les tumeurs étaient classées T2 dans la majorité des cas (65,38%). Les ganglions régionaux ont été classés N0 chez la majorité des patientes (61,53%). Les analyses génomiques du CSTN ont permis d´adapter des conduites thérapeutiques d´une manière efficace. Toutefois, il n'y a pas encore un test diagnostique établi ou vérifié cliniquement pour la classification de CSTN et donc une standardisation d´une approche thérapeutique claire.

## Abstract (en)

Triple-negative breast cancer (TNBC) is a very heterogeneous disease and refers to a subgroup of breast cancer. The purpose of this study was to describe the clinical, pathological and molecular features of TNBC. We conducted a retrospective analysis of 56 cases of TNBC diagnosed in the Department of Pathology at the Farhat Hached University Hospital over a period of three years (2018-2020). The incidence of TNBC was 5.62% and the mean age of the patients was 50.36 years. The most frequent reason for consultation was the discovery of breast nodules (98.21%). The diagnosis of TNBC was confirmed in 24 cases (42.86%) based on the analysis of mastectomy specimens. Invasive carcinoma of no special type (NST) was the most common histological type (47 cases; 82.5%). Mean tumor size was 35.5 mm. SBR grading was only specified in 42 cases (73.68%). Grade III was the most predominant type (78.6%, n = 33). The notion of lymph node metastasis was found in 44 reports and lymph node invasion was reported in 19 patients (43.2%). Immunohistochemical study showed that all patients were hormone receptor-negative (RO and RP) with no Her2 overexpression (n = 56; 100). This study results are generally in agreement with literature data. Despite recent molecular classifications, there is no clinically verified diagnostic test allowing for clear therapeutic standardization.

## Introduction

Le cancer du sein est un véritable problème de santé publique et représente le cancer le plus fréquent chez la femme à l´échelle mondiale et la première cause de mortalité féminine par cancer [1]. En Tunisie, son incidence augmente de plus en plus avec un taux de 2000 nouveaux cas diagnostiqués chaque année [1,2]. Au cours ces dernières années, la classification et la compréhension de la carcinogenèse mammaire ont progressé grâce aux analyses des profils d´expression transcriptomique des carcinomes infiltrants du sein permettant ainsi une adaptation des conduites thérapeutiques d´une manière efficace [3-6]. Le cancer du sein triple négatif (CSTN) est un sous-groupe hétérogène des cancers du sein. Il est défini par l'absence d'expression des récepteurs hormonaux (RO et RP) et de la surexpression de Her2 par immunohistochimie, limitant ainsi les options thérapeutiques ciblées. C'est un groupe hétérogène de tumeurs avec différents portraits moléculaires et pronostiques. Une classification récente du CSTN individualise quatre sous-types spécifiques (CSTN type-4) en se basant sur les données d'expression géniques et qui sont les suivants: basal-like 1 et basal-like 2 (qui diffèrent par la réponse immunitaire), mésenchymateux, et luminal AR (androgen Receptor). Ces sous-types présentent un taux de survie ainsi qu´une sensibilité à la chimiothérapie néoadjuvante différents [6]. Toutefois, malgré les multiples efforts déployés, il n'y a pas encore un test diagnostique établi ou vérifié cliniquement pour la classification de CSTN. Les perspectives actuelles visent à identifier des cibles thérapeutiques spécifiques en se basant sur les études génomiques afin d´améliorer le pronostic de ce type de cancer. L´objectif de ce travail rétrospectif était d´étudier les caractéristiques anatomo-cliniques et moléculaires du CSTN et les comparer aux données de la littérature.

## Méthodes

**Type d´étude**: il s´agit d´une étude rétrospective exhaustive portant sur 56 cas de cancer du sein triple négatif diagnostiqués au service d´Anatomie et Cytologie Pathologiques du Centre Hospitalo-Universitaire Farhat Hached de Sousse, durant une période de trois ans allant de janvier 2018 jusqu´à décembre 2020.

**Recueil des données**: le recueil des données était effectué à partir des comptes rendus anatomo-pathologiques des patients. Tous les cas de cancer du sein triple négatif diagnostiqués pendant la période de notre étude ont été inclus: Les patients (de sexe féminin et masculin) ayant un cancer du sein confirmé par l´histologie pendant la période de l´étude avec: Récepteur hormonaux négatifs: RO (-); RP (-) et pas d´expression ni amplification Her2.

**Etude statistique**: les données de cette étude ont été saisies et analysées à l´aide du logiciel d´étude statistique d´IBM: SPSS version 22.0.

**Considérations éthiques**: les différents participants dans ce travail déclarent ne pas avoir de conflit d´intérêt avec cette étude. L´anonymat et la confidentialité ont été respectés dans toutes les étapes de traitement des données.

## Résultats

**Caractéristiques générales**: durant une période de trois ans allant de 2018 à 2020, 996 patients étaient diagnostiqués de cancer du sein. Parmi ces patients, 56 femmes étaient atteintes de cancer du sein triple négatif CSTN, soit une fréquence de 5.62% du nombre global de cancer du sein. Tous les patients diagnostiqués de CSTN étaient de sexe féminin (n=56 ;100%). L´âge moyen de nos patientes était de 50,36 ans avec des âges extrêmes de 23 à 81 ans. La tranche d´âge de 35 à 40 ans était la plus touchée.

**Caractéristiques cliniques et paracliniques**: la découverte d´un nodule du sein était le motif de consultation le plus fréquent dans cette série avec 55 cas soient 98,21% et un seul cas d´adénopathie axillaire soit 1,79%. Concernant les examens para cliniques, les anomalies mammographiques ont été classées selon la classification ACR (American College of Radiology). Cette classification n´a pas été précisée que pour 24 cas (42,86%). Ces anomalies ont été classées ACR 4 dans 11 cas (45,83%) et ACR 5 dans 11 cas (45,83%) et ACR 3 dans 2 cas (8,34%). Le diagnostic de CSTN était documenté dans 24 cas (42,86%) sur des pièces de mastectomie, dans 21 cas (37,5%) sur des pièces de tumorectomie, dans 7 cas (12,5%) sur des biopsies, dans deux cas (3,56%) sur des pièces de zonectomie, dans un seul cas (1,79%) sur une pièce de quadrantectomie et dans un seul cas (1,79%) sur une pièce d´adénectomie.

**Figure 1:**
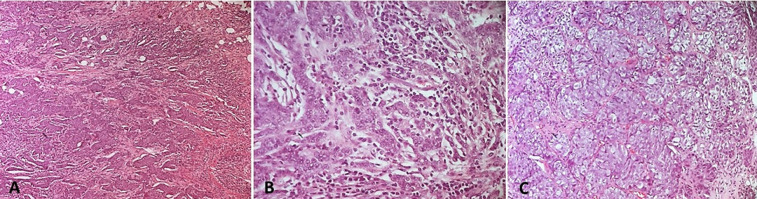
carcinome infiltrant de type non spécifique; A) (HE x 10): prolifération carcinomateuse infiltrante faite essentiellement d’amas et de travées souvent anastomosés avec un stroma fibreux d’abondance moyenne et modérément inflammatoire; B) (HE x 10): les cellules tumorales sont de taille moyenne et présentant des atypies nucléaires modérées avec un index mitotique élevé; C) carcinome infiltrant avec des aspects médullaires; il est fait d’amas de taille variable avec des plages d’aspect syncytial au sein d’un stroma lymphocytaire modérément abondant (les cellules tumorales montrent des atypies nucléaires marquées et de nombreuses mitoses (HEx200))

**Figure 2:**
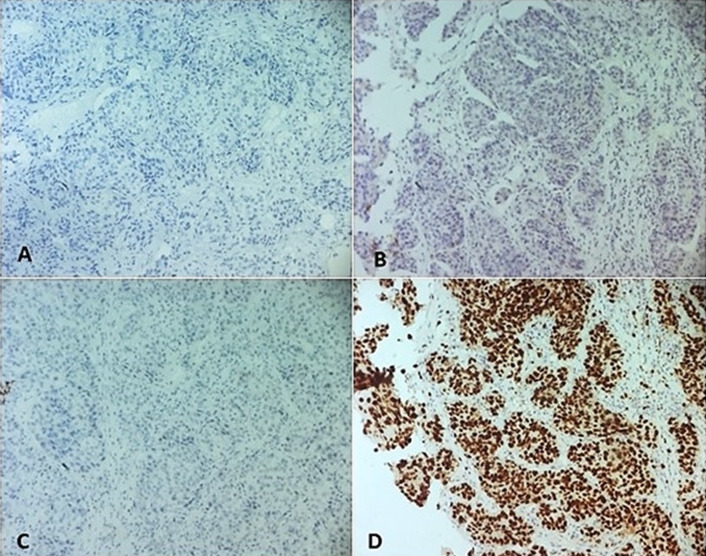
l’étude immunohistochimique montre une absence d’expression des récepteurs hormonaux; les récepteurs d’œstrogènes (RO) (A, x100) et les récepteurs de progestérone (RP) (B, x100) et une absence de surexpression de du récepteur Her2 (C, x100); l’index de prolifération Ki 67 est estimé dans ce cas à 80% (D, x100)

**Figure 3:**
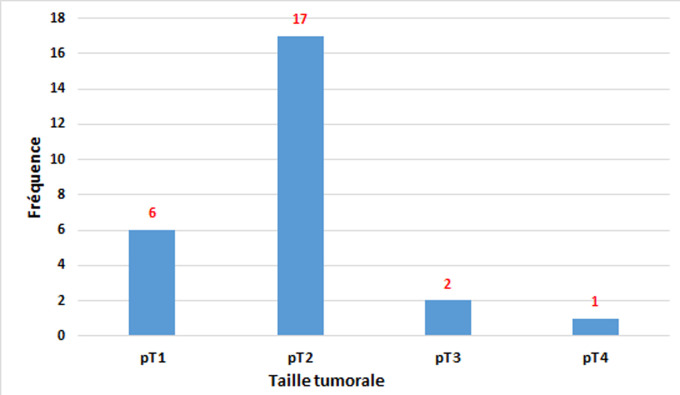
répartition selon la taille tumorale

**Caractéristiques histologiques et moléculaires**: le type histologique le plus fréquent était le carcinome infiltrant de type non spécifique dans 47 cas soit 82,5 % (Figure 1, A-B), suivi de carcinome infiltrant avec différenciation apocrine dans 3 cas (5.27%) et carcinome infiltrant avec des aspects médullaires dans 3 cas (5.27%) (Figure 1, C) puis carcinome infiltrant micropapillaire dans 2 cas (3,5%). La taille tumorale moyenne était de 35,5 mm avec des extrêmes de 2 et 120 mm. Le grade histopronostique de Scarff-Bloom-Richardson (SBR) n´était précisé que chez 42 de nos patientes (73,68%). Le grade SBR III était le plus prédominant dans notre étude (78,6%). Le grade SBR II était trouvé dans 19% des cas et le grade I dans 2,4% des cas. La recherche d´emboles vasculaires était notée dans 55 comptes rendus dont 14 cas se sont avérés positives (25,45%). La notion de métastase ganglionnaire était notée dans 44 comptes rendus. L´envahissement ganglionnaire était retrouvé chez 19 patientes, soit 43,2%. A l´immunohistochimie, tous les cas de notre série montrent une absence d´expression des récepteurs hormonaux: les récepteurs d'œstrogènes (RO) et les récepteurs de progestérone (RP) (n=56 ;100%) (Figure 2, A-B). Tous les cas de notre série montrent une absence de surexpression du récepteur Her2 dans tous les cas avec un score 0 (négatif) mentionné dans 83,93% des cas (Figure 2, C), le score 1 (négatif) dans 14,29% des cas et le score 2 (équivoque) dans 1,78% des cas. Dans ce dernier cas, une étude par hybridation chromogénique *in situ* (CISH) était réalisée et objectivant une absence d´amplification de l´oncogène HER2. L´index de prolifération Ki 67 était évalué dans 55 cas dont 7 avait un taux ≤ 14% alors que 48 cas avaient un taux > 14% (Figure 2, D). A l´issu de ces résultats immuno-histochimiques, tous les cas de cancer (100%, n=56) avaient un profil moléculaire triple négatif. La notion de chimiothérapie néoadjuvante était mentionnée dans 12 cas soit 21,43%. Les répartitions des tumeurs selon la taille tumorale et le statut ganglionnaire sont résumées dans la Figure 3 et le Tableau 1. A l´issue de l´étude anatomopathologique, la classification pTNM a pu être établie. Le statut M (métastase à distance) n´était pas mentionné dans tous les comptes rendus. La classification de Sataloff (post chimiothérapie néoadjuvante) était adressée dans 12 cas.

**Tableau 1:** répartition selon le statut ganglionnaire

Variables	Fréquence	Pourcentage(%)
**N0**	16	61,53
**N1**	6	23,08
**N2**	1	3,85
**N3**	1	3.85
**Nx**	2	7,69

## Discussion

Les définitions clinicopathologiques et moléculaires des sous-types du carcinome du sein invasif ont été adoptées par la 13^e^ conférence internationale du groupe d'experts sur le cancer du sein de Saint-Gall (2013) qui est basé sur des critères immunohistochimiques intéressant les RO, RP, ERBB2 (HER2) et Ki-67 avec confirmation d'hybridation *in situ* dans le cas échéant [7]. Ces sous-types sont Luminal A-like, Luminal B-like (HER2-négatif), Luminal B-like (HER2-positif), HER2-positif (non-luminal) et Triple-négatif. En 2011, Lehmann *et al*. ont effectué un profilage d´expression génique sur des échantillons de tumeurs chez 587 patientes ayant un CSTN. Cette étude a classé les CSTN en six sous-types: basal-like 1 (BL1), basal-like 2 (BL2), mésenchymateux (M), mésenchymateux stem-like (MSL), immuno modulateur (IM) et luminal récepteur aux androgènes (LAR) [8]. Cette nouvelle classification a permis de fournir un modèle cellulaire précis pour adapter la conduite thérapeutique du CSTN.

L´incidence du CSTN était selon notre étude 5,62%. Ce cancer représente 10 à 25% des cancers du sein infiltrants selon les séries publiés dans la littérature [9]. La prévalence de ce type de cancer varie en fonction des races et parait plus élevée chez les femmes afro-américaines que les femmes caucasiennes [10] avec un âge souvent inférieur à 50 ans, voire moins de 40 ans au moment du diagnostic [10]. D´après notre étude, la tranche d´âge de 35 à 40 ans était la plus touchée. Ainsi, les femmes âgées entre 35 et 40 ans sont à plus haut risque de CSTN. Ce fait constitue un indicateur donc de susceptibilité génétique. Le CSTN est fréquemment associé à une histoire familiale de cancer du sein. La prédisposition héréditaire à ce type de cancer est essentiellement liée à la mutation du gène BRCA1 [11-15]. La confirmation diagnostique du cancer du sein est adressée par l´examen histologique. Sur le plan histologique, le CSTN est hétérogène, de type carcinome infiltrant de type non spécifique dans la majorité des cas. Cette forme représente 70 à 80% des cancers infiltrants [16]. Néanmoins, il est important de souligner que certains CSTN correspondent à des types histologiques spéciaux, dont certains sont d´excellent pronostic comme le carcinome adénoïde kystique et le carcinome sécrétant, souvent appelé carcinome sécrétant juvenile [17,18]. Le carcinome médullaire est l´un des types de CSTN qui est considéré comme de bon pronostic malgré son caractère hautement prolifératif et faiblement différencié [18]. Les carcinomes métaplasiques appartiennent au CSTN et sont de plus mauvais pronostic, notamment en cas de haut grade [18]. Le carcinome avec différenciation apocrine, qui exprime le « androgen receptor » (AR) et le carcinome à cellules acinaires sont également des CSTN [18]. Dans notre étude le type histologique était dominé par le carcinome infiltrant de type non spécifique dans 47 cas soit 82,45 %.

Les métastases ganglionnaires sont observées dans plus de moitié des cancers du sein lors du diagnostic [19-21]. Dans notre série, l´envahissement ganglionnaire était retrouvé chez 43,2% des cas. A l´immunohistochimie, une forte expression de Ki67 est associée généralement à un mauvais pronostic. Toutefois, ce taux élevé observé dans la majorité des CSTN est prédictif d´une bonne réponse à la chimiothérapie néoadjuvante [22]. Sur le plan thérapeutique, la chirurgie reste le traitement de choix, associé à un traitement adjuvant locorégional par radiothérapie, et un traitement systémique par chimiothérapie. Le CSTN est habituellement chimiosensible en raison de l´index mitotique élevé, avec un taux important de rémission histologique complète en situation néoadjuvante [23]. L´hétérogénéité de la réponse à la chimiothérapie reflète l´hétérogénéité et la complexité moléculaire du CSTN qui inclut différentes entités intrinsèques, basal-like et non basal-like [24]. D´où l´intérêt de phénotyper ce type de cancer afin d´adapter la chimiothérapie.

Compte tenu du grand nombre d´altérations moléculaires dont certaines pourraient représenter des cibles thérapeutiques potentielles, un certain nombre de thérapies ciblées ont été évaluées et font l´objet d´essais cliniques, tels que les inhibiteurs de PARP (poly (ADP-ribose) polymérase) et d´EGFR, les agents anti-angiogéniques et les anti-androgènes [24-25]. L´immunothérapie représente également l´une des nouvelles alternatives thérapeutiques du CSTN. Notre compréhension de l'interaction complexe entre le cancer et le système immunitaire s'est considérablement améliorée en passant du concept de « surveillance immunitaire » à celle de “immunoediting” [26]. Un exemple d'immunoediting tumoral dans le CSTN est fourni par la présence de mutations CASP837, qui peuvent abroger la mort induite par les lymphocytes T CD8 + cytolytiques, et a été décrit comme un mécanisme commun d´échappement immunitaire dans de nombreuses tumeurs solides [27]. Malgré leur chimiosensibilité, le CSTN est de moins bon pronostic par rapport aux autres groupes en raison d´une progression rapide chez les non-répondeurs. Les principales limites de notre travail étaient essentiellement, le caractère rétrospectif de l´étude qui ne permettait pas d´évaluer d´une manière objective l´évolution clinique et la survie post thérapeutique et le l´échantillon réduit de l´étude. Notre travail a permis, en revanche de mettre le point sur les principales caractéristiques épidémiologiques et clinico-pathologiques du CSTN ainsi que la revue de la littérature concernant le profil moléculaire et l´approche thérapeutique récente.

## Conclusion

Les cancers du sein triple négatifs (CSTN) représentent un sous-groupe de cancers du sein très hétérogène sur les plans clinique, morphologique, moléculaire et pronostique. Les résultats de notre étude rejoignent les données de la littérature concernant les caractéristiques épidémiologiques et clinico-pathologiques du CSTN. Malgré les multiples efforts déployés, il n'y a pas encore un test diagnostique établi ou vérifié cliniquement pour la classification de CSTN et donc une standardisation d´une approche thérapeutique claire. Cela nécessite alors une collaboration internationale impliquant les anatomopathologistes, les oncologues et chercheurs fondamentaux afin de valider ces tests diagnostiques basés sur les signatures moléculaires et les thérapies ciblées.

### Etat des connaissances sur le sujet

- Le cancer du sein triple-négatif (CSTN) représente un sous-groupe de cancers du sein très hétérogène;
- La chimiothérapie néoadjuvante est le seul traitement systémique validé;
- La compréhension de la carcinogenèse mammaire a progressé grâce aux analyses des profils d´expression transcriptomique.

### Contribution de notre étude à la connaissance

- Le profilage d´expression génique des CSTN a permis une adaptation des conduites thérapeutiques d´une manière efficace;
- Malgré les multiples efforts déployés, il n'y a pas encore un test diagnostique établi ou vérifié cliniquement pour la classification de CSTN.
